# Topiramate-Induced Aqueous Misdirection in a Nanophthalmic Eye

**DOI:** 10.7759/cureus.36529

**Published:** 2023-03-22

**Authors:** Ahsan Hussain, Kiersten Snyder, Shahnawaz Paroya, Sankara Mahesh

**Affiliations:** 1 Ophthalmology, Boston University Chobanian & Avedisian School of Medicine, Boston, USA; 2 Ophthalmology, New York Medical College, Valhalla, USA; 3 Biology, The University of Kansas, Kansas City, USA

**Keywords:** ophthalmology, ciliary body, angle closure, glaucoma, topiramate

## Abstract

A 51-year-old female was referred to the emergency department with a one-day history of severe right eye pain, blurry vision, and conjunctival injection. A review of past ocular history was notable for nanophthalmos and narrow angles with patent peripheral iridotomies. Anterior segment exam findings were consistent with aqueous misdirection and a review of medications indicated recent topiramate initiation for headaches and depression. The acute attack was initially controlled with medical management and plans for future surgical intervention were made. Although ocular screening prior to initiation of topiramate is not recommended, this case highlights the importance of pre-screening in a patient with a pre-existing condition such as nanophthalmos. Additionally, this case addresses the ocular side effects of anti-depressants and the emerging relationship between glaucoma and depression. Appropriately addressing these issues and coordinating care with behavioral health providers has the potential to prevent optic nerve damage and loss of vision.

## Introduction

Topiramate is an anti-epileptic drug that has been used off-label for the treatment of migraines, as well as some mood disorders including biopolar disorder and depression. One of the known side effects of topiramate is acute angle-closure glaucoma thought to be caused by ciliochoroidal effusion with forward displacement of the lens-iris diaphragm and anterior chamber shallowing, resulting in acute myopia and angle-closure glaucoma [1]. We present the first reported case of topiramate-induced angle-closure glaucoma in a nanophthalmic eye. Although ocular screening prior to initiation of topiramate is not recommended, this case highlights the importance of pre-screening in a patient with a pre-existing condition such as nanophthalmos, which is often associated with varying degrees of angle-closure glaucoma.

## Case presentation

A 51-year-old female with a past medical history of depression and migraine presented to the emergency department with a one-day history of severe right eye pain, blurry vision, and conjunctival injection. Past ocular history was notable for nanophthalmia in both eyes with axial lengths of 17.29 right eye (OD) and 16.96 left eye (OS). On an earlier exam, the patient’s best-corrected visual acuity was 20/50 with a refractive error of +9.00 diopters in both eyes. The patient had narrow angles and had undergone prophylactic laser peripheral iridotomies (LPIs). She was seen in the neurology clinic one month prior to this presentation and was started on topiramate 25mg for her migraine headaches and depression.

**Figure 1:**
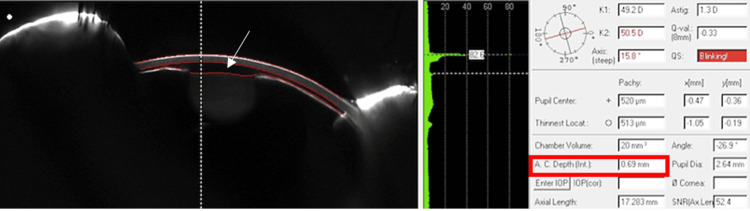
Pentacam photograph of the right eye demonstrating narrow angles and a severely shallow anterior chamber with a depth of .69mm.

The patient’s best-corrected visual acuity was counting fingers in the right eye and 20/50 in the left eye. Her intraocular pressure (IOP) was 65 and 12 in the right and left eye, respectively. Anterior segment exam showed 3+ conjunctival injection and mild corneal edema with a severely narrow anterior chamber depth of 0.69 mm (Figure 1).

In addition, there was iridocorneal touch and 360 degrees of peripheral anterior synechiae with anterior displacement of the lens-iris diaphragm. The posterior segment exam showed a cup to disc ratio of 0.2 with normal appearing fovea and peripheral retina. In the setting of elevated intraocular pressure, anterior chamber shallowing, with a patent peripheral iridotomy, her exam findings were consistent with aqueous misdirection likely due to ciliary effusion and rotation of the ciliary body from topiramate [1]. The offending agent was subsequently discontinued and she was treated to lower the IOP with intravenous acetazolamide, intravenous mannitol, and multiple rounds of topical glaucoma medications, including dorzolamide, timolol, and brimonidine. A cycloplegic was administered to facilitate posterior rotation of ciliary body and lens-iris diaphragm. After stabilizing her IOP, a vitrectomy and lensectomy were performed to provide definitive management.

## Discussion

Topiramate-induced secondary angle-closure glaucoma is a rare side effect of a common medication. Despite initial approval as an anti-epileptic drug, neurologists and psychiatrists frequently prescribe this agent for the treatment of migraine headaches, depression, and bipolar disorder. This drug is well-documented to cause anterior rotation of the ciliary body and decrease zonular tension with forward displacement of the lens [2]. The pathogenesis of this aqueous misdirection is likely due to excess fluid accumulation in the ciliary body and choroid due to the increased permeability of ciliochoroidal vasculature [3]. Routine ocular screening prior to starting topiramate is not traditionally recommended. However, a nanophthalmic eye has a higher baseline risk for angle closure due to a narrow anterior chamber, as well as an increased risk of developing uveal effusions due to the thickening of the sclera. Scleral thickening of up to twice the normal depth with atypical collagen fibrils results in the obstruction of venous vortex drainage and a decreased uveoscleral outflow [4,5]. In our case, a crowded anterior segment, baseline risk of uveal effusions, and simultaneous use of Topiramate fashioned the perfect storm for angle closure.

Initial management of aqueous misdirection involves lowering the intraocular pressure with aqueous suppressants, employing cycloplegic drops to aid in posterior rotation of the ciliary body and lens, and using hyperosmotic agents to reduce vitreous volume. In the early stage of glaucoma, laser peripheral iridotomy (LPI) can help eliminate the pupillary block before the occurrence of peripheral anterior synechiae develops [6]. While the initial treatment is through medical management, studies show a recurrence rate as high as 100% with medical therapy alone [7]. Definitive treatment includes surgical and can involve a lensectomy and vitrectomy to create a unicameral eye. Operating with a shallow or flat anterior chamber in nanophthalmic eyes presents a unique challenge even in the hands of skilled ophthalmologists. A shallow anterior chamber creates a difficult environment to perform capsulorhexis and routine maneuvers without damaging intraocular structures. Decompressing the vitreous through a surgical vitrectomy can greatly aid in increasing anterior chamber size and decrease positive pressure prior to phacoemulsification [7,8]. Other surgical treatments that have been advocated include irido-zonulo-hyaloidectomy with or without a vitrectomy [9]. Additional considerations include creating scleral windows to enhance uveoscleral outflow and to decrease the potential for uveal effusions and expulsive choroidal hemorrhage during cataract surgery.

Though this case demonstrates the association between topiramate and aqueous misdirection, it also highlights the pressing relationship between glaucoma patients and clinical depression. Glaucoma can be a debilitating disease that significantly impacts the quality of life of an individual. Recent studies have consistently demonstrated the alarmingly high rate of depression in glaucoma patients [10-13]. Studies show as high as 57% of patients with primary open-angle (POAG) meeting clinical parameters for depression and glaucoma patients face a 1.7x increased risk of developing depression [9,11]. Moreover, the likelihood of clinical depression is positively correlated with glaucoma severity [14]. Stamatiou et al. performed a systematic review of the literature and found certain risk factors such as age, female gender, advanced-stage disease, faster visual loss progression, and the lifestyle of patients were proven to increase the prevalence of depression in glaucoma patients [15].

This unfortunate reality needs to be recognized and care must be coordinated with the patient’s behavioral health provider. While topiramate is believed to induce ciliochoroidal swelling and anterior movement of the lens, other antidepressant medications can cause primary angle closure through alternative mechanisms. The current standard of treatment for clinical depression involves initiating a selective serotonin-reuptake inhibitor (SSRI). This class of medications can trigger iris dilation and induce pupillary block through anticholinergic properties. Patients with narrow angles should prophylactically receive LPIs prior to starting SSRI, and high-risk eyes such as nanophthalmic eyes should not be prescribed an angle-closure-provoking medication. A careful review of systemic medications and medical history by the ophthalmologist can help coordinate care with the patient’s neurologists and behavioral health providers [13]. An open dialogue regarding mental health in glaucoma patients and appropriate referral to qualified professionals needs to be implemented routinely to not only coordinate care for the patient’s eyes but overall well-being.

## Conclusions

We present the first reported case of topiramate-induced angle-closure glaucoma in a nanophthalmic eye. In addition to reviewing the management of aqueous misdirection, our case highlights the emerging relationship between glaucoma and clinical depression. As the preponderance of contemporary anti-depressive agents can induce angle closure glaucoma, careful attention must be placed on high-risk patients and treatment needs to be coordinated with the patient’s mental health provider.
